# The Prognostic Role of SIRT1-Autophagy Axis in Gastric Cancer

**DOI:** 10.1155/2016/6869415

**Published:** 2016-12-14

**Authors:** Guanglin Qiu, Xuqi Li, Chao Wei, Xiangming Che, Shicai He, Jing Lu, Shufeng Wang, Ke Pang, Lin Fan

**Affiliations:** ^1^Department of General Surgery, The First Affiliated Hospital Medical College of Xi'an Jiaotong University, Xi'an 710061, China; ^2^Xi'an Health School, Xi'an 710054, China; ^3^Shaanxi Friendship Hospital, Xi'an 710068, China

## Abstract

*Aim*. Sirtuin 1 (SIRT1) can induce autophagy through deacetylation of Beclin-1 and other autophagy mediators. However, the relationship between SIRT1 and autophagy in GC has not been defined. Therefore, the aim of this study was to confirm the prognostic value of SIRT1 and Beclin-1 and their relationship in GC patients.* Methods*. Transmission electron microscopy (TEM) was performed to examine the autophagy in GC patients. Immunohistochemistry was used to examine the expression of SIRT1, Beclin-1 in GC, and adjacent nonneoplastic mucosa.* Results*. In 7 out of 8 GC patients' samples examined by TEM, more autophagic vesicles were observed in GC tissues compared to adjacent nonneoplastic mucosa tissue. A positive correlation between SIRT1 and Beclin-1 expression was observed. Furthermore, Beclin-1 or SIRT1 expression alone or their combined expression were significantly correlated with advanced clinicopathological parameters. High Beclin-1 and SIRT1 expression alone and their combined high expression predicted shorter overall survival and relapse-free survival. Both high Beclin-1 and SIRT1 expressions were independent prognostic factors for poor survival of GC.* Conclusions*. Based on our results we can conclude that SIRT1 and Beclin-1 expression alone or in combination can be used as prognostic indicator and may represent new therapeutic targets in GC.

## 1. Introduction

Gastric cancer (GC) is the fourth most common cancer and the third leading cause of cancer-related deaths worldwide [1]. Due to the absence of specific symptoms, as well as the lack of reliable methods for early detection, the majority of patients with GC are diagnosed at advanced stages, and the 5-year relative survival rate is less than 28% [2]. Although much is known about the risk factors, pathogenesis, and clinical features of GC, specific molecular markers with predictive and prognostic value, as well as, potential therapeutic targets still remain limited.

Autophagy is a cellular degradation and recycling process by which cells dispose of superfluous or nonfunctional components. In brief, these components are sent to the lysosome for degradation and new substrates for energy generation and biosynthesis are generated through this process [3]. It has been shown that autophagy may have a dual role depending upon specific physiological conditions. For example, autophagy can result in cell survival or can induce cell death during tumorigenesis and/or therapy [4]. In GC, anticancer drugs, which induce autophagy, can both suppress [5] or promote tumor cell growth [6]. However, the role of autophagy in the progression of GC is still poorly understood.

Beclin-1 was the first identified mammalian autophagy-related protein and is an essential component of the process involved in cell death and cell survival [7]. Indeed, it has been used in many studies as a marker to monitor autophagy [8, 9]. High expression of Beclin-1 has been reported in ovarian cancer [10], GC [11, 12], nasopharyngeal carcinoma [13], intrahepatic cholangiocarcinoma [14], while low expression has been reported in hepatocellular carcinoma [15] and colorectal cancer [16]. Furthermore, high Beclin-1 expression has been shown to be a poor prognostic factor in ovarian cancer [10], nasopharyngeal carcinoma [13], but a favorable prognostic factor in GC [11, 12], intrahepatic cholangiocarcinoma [14], hepatocellular carcinoma [15] and colorectal cancer [16]. Nevertheless, the exact role of Beclin-1 in GC tumorigenesis is still unclear.

Sirtuin1 (SIRT1) is a NAD^+^-dependent deacetylase, mainly deacetylating histones and many nonhistone targets, including p53, FoxO1, FoxO3, Atg5, Atg7, Atg8, Ku70, NF-*κ*B, and PTEN, and is involved in tumor development, energy homeostasis, autophagy, DNA damage repair, and other important cellular processes [17]. Several reports have shown that SIRT1 expression is a prognostic indicator for many cancers including GC [18–20]. In addition, it has been shown that SIRT1 plays a role in cellular proliferation, epithelial-mesenchymal transition (EMT), cell invasion, and chemoresistance in GC cells and, therefore, may be considered a potential therapeutic target [21, 22]. However, the exact relationship between SIRT1 expression and GC progression is not yet conclusive.

Recently, the importance of acetylation in autophagy control has been established. It has been reported that SIRT1 can regulate autophagy through the deacetylation of Atg5, Atg7, Atg8, and other autophagy mediators, which in turn plays an important role in the regulation of proliferation, metabolism, and stress resistance in different cells [17]. Furthermore, Beclin-1 modifications may lead to the inhibition, fine-tuning, or induction of the autophagic response under different cellular conditions. For example, it has been reported that Beclin-1 is acetylated by p300 and deacetylated by SIRT1 at lysine residues 430 and 437 which further influences the autophagosome maturation and tumor growth [23].

Despite these findings, the exact role of the SIRT1-autophagy axis in GC has not been identified. Therefore, in this study, we examined autophagy in fresh GC specimens by using transmission electron microscopy (TEM). In addition, we examined the expression and prognostic properties of Beclin-1 and SIRT1 by immunohistochemistry in paraffin embedded human GC tissue, as well as in surrounding nonneoplastic mucosa (NNM).

## 2. Materials and Methods

### 2.1. Patients and Samples

Paraffin sections of GC tissue (*n* = 96) and adjacent NNM (*n* = 96) (at least 10 cm away from the tumor tissue) were taken from 96 patients, who were treated with curative tumor resection in the First Affiliated Hospital Medical College of Xi'an Jiaotong University (Xi'an, China) between January 2008 and December 2008.

The clinicopathological parameters which were reported included gender, age, body mass index (BMI), tumor size, tumor location, Lauren classification, histological grade, lymph node (LN) metastasis, depth of invasion (early gastric carcinoma versus advanced gastric carcinoma), tumor node metastasis (TNM) stage (reviewed based on the TNM staging system of the American Joint Committee on Cancer [24]) (I and II versus III and IV), vascular cancer embolus (VCE), patient survival, Beclin-1 expression level, and SIRT1 expression level.

All patients were selected according to the following criteria: (a) primary GC diagnosis without any other concomitant malignancy, (b) patients received neither chemotherapy nor radiotherapy before tumor resection, (c) the follow-up was done immediately after the surgical treatment, (d) detailed clinicopathological data and follow-up records were available. In addition, we also collected fresh GC tissue and adjacent NNM from the surgical resection of 8 patients with GC during October 2013 for the TEM assay. Patient age ranged from 27 to 74 years (median, 51.7 ± 15.0 years), 5 were male and 3 were female, and the other clinicopathological data was shown in Table S1 in Supplementary Material available online at http://dx.doi.org/10.1155/2016/6869415. All 8 patients were selected according to the following criteria: (a) primary GC without any other concomitant malignancy and (b) receiving neither chemotherapy nor radiation therapy before tumor resection.

The mean follow-up time of 96 GC patients was 31.6 months (range, 6 to 78 months). Of 96 patients included in the study, 59 patients had died, 14 patients have been lost to follow-up, and 23 patients were still alive during the study period. Therefore, the lost to follow-up rate in our study was 14.58%. The median time to death was 14.0 months (range: 6 to 56 months). Patients were followed up by consulting their case documents and by telephone interview until death or December 2014. Overall survival (OS) was defined as the length of time from surgery treatment to death or last contact due to any cause. Relapse-free survival (RFS) was defined as the time from surgery treatment to cancer recurrence, distant metastasis, death, or last contact.

Formalin fixed paraffin embedded sections of GC tissue (*n* = 96) and adjacent NNM (*n* = 96) were taken from 96 patients for immunohistochemical staining. For the TEM assay, fresh GC tissue and adjacent NNM from the surgical resection of 8 patients with GC were used. The usage of both sample groups has been permitted by the Institutional Review Board at the First Affiliated Hospital Medical College of Xi'an Jiaotong University (Xi'an, China) and the study conformed to ethical guidelines of 1964 Declaration of Helsinki. Written informed consent was obtained from both groups of patients: 96 patients which were recruited between January and December of 2008 and 8 patients were selected in 2013.

### 2.2. Transmission Electron Microscopy

For TEM analysis, fresh tissue from the surgical resections of 8 patients with GC was fixed immediately in 2.5% glutaraldehyde solution at room temperature and sent for TEM analysis (including sample fixation, rinsing, postfixation, dehydration, embedding, ultra-microtomy, staining and measurement) which was provided by the Department of Electron Microscopy in the Medical College of Xi'an Jiaotong University (Xi'an, China). Sections were imaged via a TEM (H-7650, Hitachi, Japan) operated at 80 kV. The scale bar is indicated at the bottom of micrograph images; a minimum of random ten fields of view in each section at one magnification were evaluated for the appearance of autophagic vesicles.

### 2.3. Immunohistochemical Staining

Immunohistochemical staining of Beclin-1 and SIRT1 was performed on a 4 *μ*m thick paraffin embedded tissue sections of GC tissue and adjacent NNM using streptavidin-peroxidase. In brief, after the deparaffinization and dehydration, tissue sections were soaked in a solution of 3% hydrogen peroxide (AR1108, BOSTER, WuHan, China) to block endogenous peroxidases and were then treated in 0.01 M sodium citrate buffer (AR0024, BOSTER, WuHan, China) in a microwave oven for antigen retrieval. Next, sections were incubated with 10% goat serum (AR0009, BOSTER, WuHan, China) to block nonspecific staining, and then sections were incubated overnight at 4°C with a monoclonal antibody against Beclin-1 (1 : 200, ab51031, clone EPR1733Y Abcam, Cambridge, USA) and a polyclonal antibody against SIRT1 (1 : 50, sc-15404, clone H-300, Santa Cruz Biotechnology, Dallas, USA). Tissue sections were then incubated with secondary anti-rabbit IgG antibody (BA1003, BOSTER, WuHan, China) at 37°C for 30 min. Visualisation of the immunohistochemical reaction was performed using 3,3′-diaminobenzidine (DAB) (AR1022, BOSTER, WuHan, China) chromogenic reaction. Nuclei were counterstained with haematoxylin (AR0005, BOSTER, Wuhan, China). Sections were then dehydrated following standard procedures and sealed with coverslips. Negative controls (GC tissue, *n* = 96; adjacent NNM, *n* = 96) were also taken from 96 patients and treated in the same manner, except that they were incubated with 0.01 M phosphate buffer saline without the primary antibody. Internal positive controls used were stromal and inflammatory cells in each section.

### 2.4. Evaluation of Immunohistochemistry

Immunohistochemical scoring was performed by two independent observers (Xiangming Che and Lin Fan) blinded to patient outcomes. Five random fields of view of every section were examined. Beclin-1 and SIRT1 expression was semiquantitatively scored by multiplying the intensity of staining and the percentage of positive cells, according to Cao et al. [25], with slight modifications. The intensity of cell staining was graded as follows: 0 (no staining); 1 (mild staining); 2 (moderate staining); 3 (intense staining). The percentage of positive cells was evaluated as follows: 0 (0-1%); 1 (1–25%); 2 (25–50%); 3 (50–75%); and 4 (>75%). Immunoreactivity score ranged within 0–12 (0, 1, 2, 3, 4, 6, 8, 9, and 12). If the final score was <4 (0, 1, 2, and 3), expression of Beclin-1 and SIRT1 was considered low; if the score was ≥(4, 6, 8, 9, and 12), the expression was considered high.

### 2.5. Statistical Analysis

Statistical analysis was performed using SPSS 19.0 (IBM SPSS, Chicago, USA) and Graph Pad Prism 5 (GraphPad Software, USA). The Pearson's *χ* ^2^ test or Fisher's exact test were used to examine the association between Beclin-1 expression, SIRT1 expression, and their combined expression alongside various clinicopathological characteristics. Multivariate logistic regression analysis was used to identify the risk factors linked with the expression of Beclin-1 and SIRT1, respectively. Pearson's *χ* ^2^ test was also used to examine the correlation between Beclin-1 and SIRT1 expression in GC and the disparity between Beclin-1 and SIRT1 expression in GC tissue and adjacent NNM tissue. The relationship between the appearance of autophagic vesicles in GC and adjacent NNM tissue was evaluated using an unpaired* t*-test. Univariate and multivariate Cox proportional hazards regression analysis was performed to evaluate the impact of clinicopathological factors and expression of Beclin-1 and SIRT1 on OS and RFS. Kaplan–Meier survival curves were constructed to further illustrate the impact of OS and RFS.* p *values of < 0.05 were considered statistically significant.

## 3. Results

### 3.1. The Autophagic Vesicles in GC and Adjacent NNM Tissues

**Figure 1:**
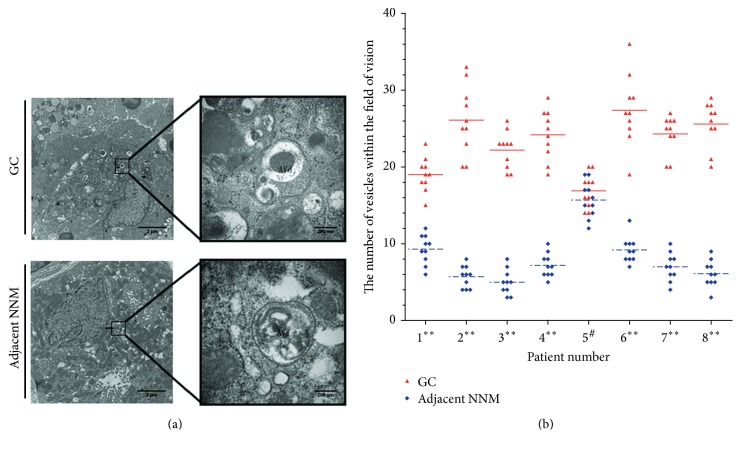
Autophagic vesicles in GC and adjacent NNM tissues. (a) Representative transmission electron microscopy images of autophagosome/autolysosome (sword) accumulation in GC and adjacent NNM tissues. A close-up of vacuolar structures revealed double membrane containing lighter content (AVi) or single membrane containing darker content (AVd). Scale bar: 2 *μ*m and 200 nm. (b) The quantification of the number of autophagic vesicles in 10 visions in each GC and adjacent NNM section. ^*∗∗*^ *p* < 0.01; ^#^ *p* > 0.05.

To determine whether the level of autophagy was different in GC compared to adjacent NNM tissues, we have examined the number of autophagic vesicles in tissues of 8 GC patients (Figure 1(a)) using TEM. Autophagosomes, also referred to as initial autophagic vacuoles (AVi), were typically identified as vacuolar structures containing cellular contents that appeared similar to the cytosol and organelles in the cell, as presented in Figure 1(a). Autolysosome, referred to as late/degradative autophagic vacuoles and autolysosomes (AVd), had higher electron density than the cytoplasm as shown in Figure 1(a). The number of autophagic vesicles was counted in at least 10 fields of view in each section at the same magnification (10,000x) (Figure 1(b)). In 7 out of 8 GC patients, a higher number of autophagic vesicles were present in GC tissue compared to adjacent NNM tissue (Figure 1(b), *p* < 0.01). These findings demonstrate that the majority of GC tissues exhibit more autophagic activity than adjacent NNM tissue.

### 3.2. Beclin-1 and SIRT1 Expression in Human GC and Adjacent NNM Tissues

**Figure 2:**
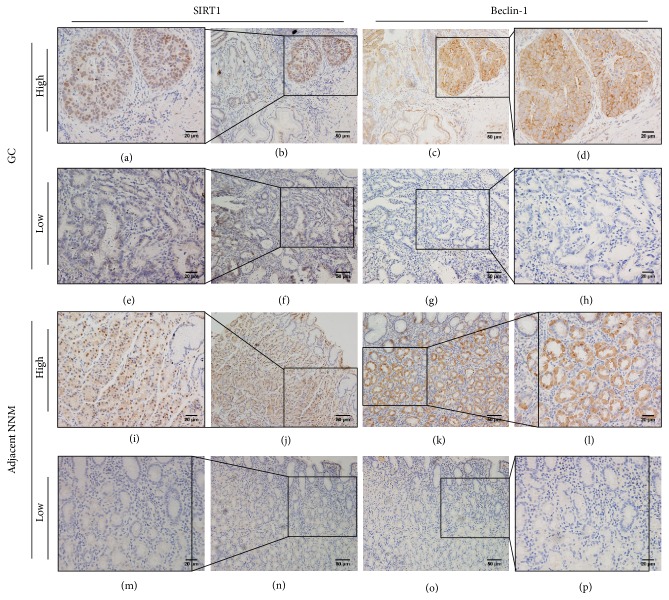
Representative images of immunohistochemical staining of Beclin-1 and SIRT1 in GC and adjacent NNM tissues. Beclin-1 is expressed in the cytoplasm of epithelial mucosal cells and GC cells. SIRT1 is expressed mainly in the cell nuclei and, to a lesser extent, in the cytoplasm of epithelial mucosal and cancer cells. Scale bar: 20 *μ*m ((a), (d), (e), (h), (i), (l), (m), (p)) and 50 *μ*m ((b), (c), (f), (g), (j), (k), (n), (o)). (a) and (d), (b) and (c): High expression of SIRT1 and Beclin-1 in GC consecutive slides and co-localization. (f) and (g), (e) and (h), (n) and (o), (m) and (p) Low expression of SIRT1 and low expression of Beclin-1 in GC and adjacent NNM consecutive slides and colocalization. (i) and (j) High expression of SIRT1 in adjacent NNM. (k) and (l) High expression of Beclin-1 in adjacent NNM.

Beclin-1 and SIRT1 protein expression were examined using immunohistochemistry. The localization of Beclin-1 was cytoplasmatic in both epithelial mucosa and GC cells. SIRT1 was expressed mainly in cell nuclei and, to a lesser extent, in the cytoplasm of epithelial mucosa and GC cells. Therefore, only nuclear SIRT1 expression was evaluated. Representative sections of positive and negative immunostaining of Beclin-1 and SIRT1 in human GC and adjacent NNM tissues are presented in Figure 2. In 24 out of 96 (25.0%) adjacent NNM tissues, and 57/96 (59.4%) cancer tissues, high expression of Beclin-1 was detected. Moreover, high SIRT1 expression was detected in 19/96 (19.8%) adjacent NNM tissues and 53/96 (55.2%) cancer tissues. Expression of Beclin-1 and SIRT1 expression between NNM and cancer tissues was found to be statistically significant (Table S2, *p* < 0.001).

**Figure 3:**
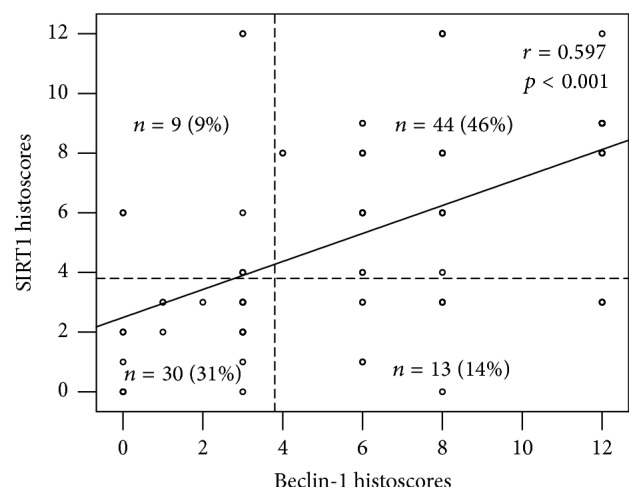
The relationship between Beclin-1 and SIRT1 expression in GC tissues. Beclin-1 expression showed a significant positive correlation with the SIRT1 expression. *r* = 0.597, *p* < 0.001.

In addition, according to the combined expression levels of Beclin-1 and SIRT1 in 96 GC tissues, we classified them into four groups as follows: Beclin-1/SIRT1 expression (low/low, *n* = 30, 31%; low/high, *n* = 9, 9%; high/low, *n* = 13, 14%; high/high, *n* = 44, 46%). The combined expression of Beclin-1 and SIRT1, using consecutive slides and colocalization is presented in Figure 2 ((a) and (d), (b) and (c), (f) and (g), (e) and (h), (n) and (o), (m) and (p), resp.). The relationship between Beclin-1 and SIRT1 expression was analyzed, and a significant positive correlation was observed (*r* = 0.597, *p* < 0.001) (Figure 3).

### 3.3. Association of Beclin-1 and SIRT1 Expression with the Clinicopathological Characteristics

The clinicopathological characteristics of GC patients are summarized in Table 1. In our study, high Beclin-1 expression was significantly correlated with tumor size (*p* = 0.001), histological grade (*p* < 0.001), LN metastasis (*p* < 0.001), TNM stage (*p* < 0.001), VCE (*p* = 0.013), and high SIRT1 expression (*p* < 0.001). High SIRT1 expression was associated with patient age (*p* = 0.046), tumor size (*p* = 0.016), histological grade (*p* = 0.007), LN metastasis (*p* < 0.001), tumor invasion (*p* = 0.015), and TNM stage (*p* < 0.001). In addition, we also analyzed the relationship between the combined expression status of Beclin-1 and SIRT1 and clinicopathological parameters. As presented in Table 1, high Beclin-1/high SIRT1 expression was also associated with tumor size (*p* = 0.002), histological grade (*p* = 0.001), LN metastasis (*p* < 0.001), tumor invasion (*p* = 0.026), TNM stage (*p* < 0.001), and VCE (*p* = 0.030). Other clinicopathological characteristics showed no statistically significant association with either Beclin-1 or SIRT1 or their combined expression (Table 1).

Moreover, clinicopathological characteristics which were found to be significantly associated with Beclin-1 and SIRT1 expression were further included into the multivariate logistic regression analysis to evaluate which characteristics have the greatest influence on their expression. Of these characteristics, large tumor size (>5 cm) (OR = 7.211, 95% CI 1.382–37.632), and high SIRT1 expression (OR = 4.617, 95% CI 1.416–15.053) were independently associated with high Beclin-1 expression, while only high Beclin-1 expression (OR = 7.818, 95% CI 1.987–30.756) was independently associated with high SIRT1 expression in GC (Table 2).

### 3.4. High Expression of Beclin-1 and SIRT1 in GC Correlates with Reduced OS and RFS

Univariate Cox proportional hazard analysis showed that large tumor size, poor histological grade, positive LN metastasis, advanced GC, high TNM stage, high SIRT1, and Beclin-1 expression were significantly related to shorter OS and RFS (*p* < 0.05) (Table 3). Patients with Beclin-1/SIRT1 high/high expression had shortest OS and RFS compared to other three groups (low/low, low/high, high/low, *p* < 0.05).

**Figure 4:**
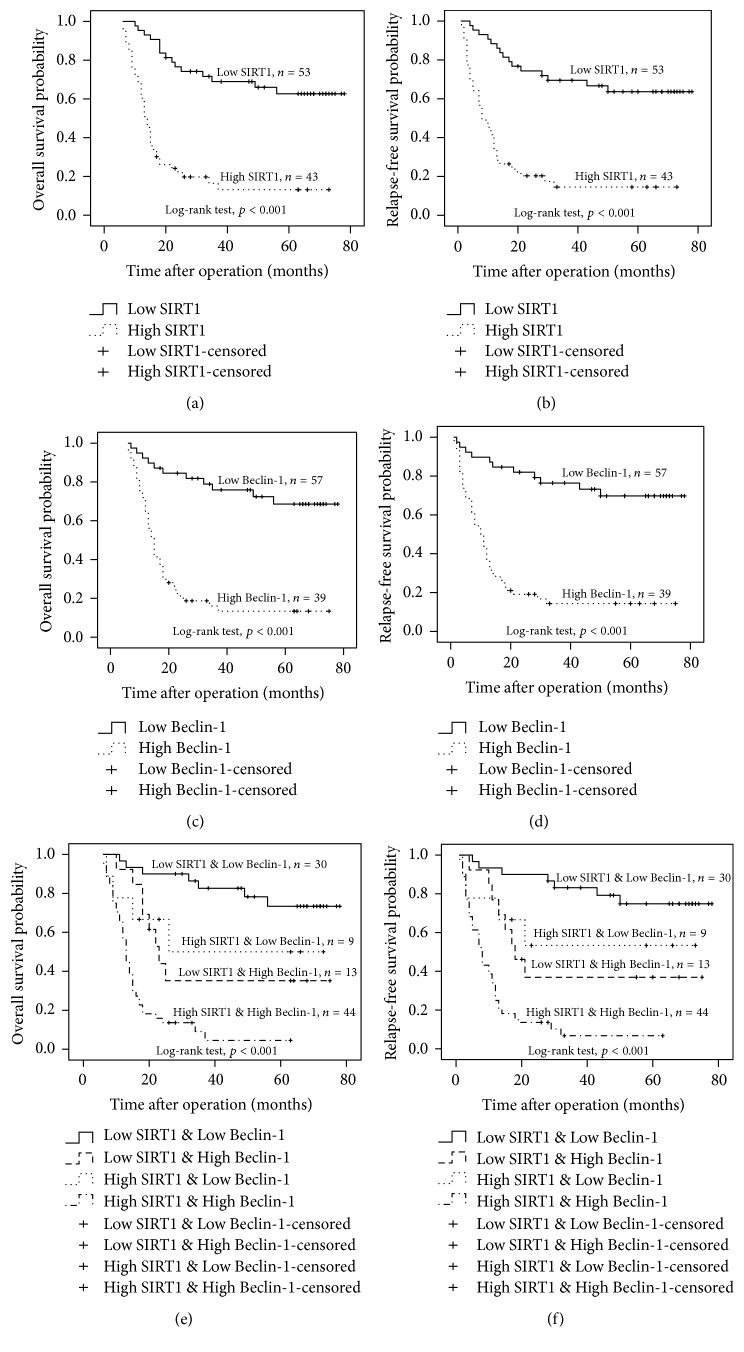
Survival analyses for Beclin-1, SIRT1, and their combined expression in GC patients. (a), (b) Association of expression level of SIRT1 with OS and RFS. (c), (d): Association of expression level of Beclin-1 with OS and RFS. (e), (f) Association of combined expression status of Beclin-1 and SIRT1 with OS and DFS.

The relationship of single or combined Beclin-1 and SIRT1 expression with OS and RFS was examined using Kaplan–Meier survival curves in Figure 4. In our study, high expression of Beclin-1 and SIRT1 was significantly correlated with shorter OS and RFS (all *p* < 0.001) (Figure 4). Patients with Beclin-1/SIRT1 high/high expression had significantly shortest OS (*p* < 0.001) and RFS (*p* < 0.001) compared to the other three groups (Figure 4). These results were consistent with the results of the univariate Cox proportional hazard analysis.

### 3.5. The Positive LN Metastasis, High Beclin-1, and SIRT1 Expression Are Independent Prognostic Factors for Poor Survival Outcome and Disease Recurrence

Clinicopathological characteristics which were found to be significantly associated with OS and RFS (tumor size, histologic grade, LN metastasis, tumor invasion, TNM stage, SIRT1 expression, and Beclin1 expression) were further included into the multivariate Cox proportional hazard analysis to evaluate which characteristics were independent prognostic factors. The results presented in Table 4 revealed that only positive LN metastasis, high Beclin-1 expression, and high SIRT1 expression were independent prognostic factors that were significantly associated with shorter OS and RFS. Patients with high Beclin-1 expression had a 2.131-fold (95% CI 1.057–4.297) greater risk for death and a 2.237-fold (95% CI 1.108–4.518) greater risk for recurrence of the disease. Patients with high SIRT1 expression had a 2.393-fold (95% CI 1.273–4.499) greater risk of death and a 2.230-fold (95% CI 1.187–4.191) greater risk for recurrence of disease.

## 4. Discussion

It has been reported that the induction of autophagy by* Helicobacter pylori* infection plays an important role in the progression of GC [17]. In GC therapy, the induction of autophagy by anti-cancer drugs, such as protocadherin 17 and vincristine, has been shown to either suppress [5] or promote tumor cell growth [6]. Nevertheless, the mechanism of these contradictory roles of autophagy in GC still remains undefined. TEM is considered a gold standard method for the evaluation of autophagy and can be utilized to qualitatively and quantitatively examine autophagy [9]. TEM has been used to detect the presence of autophagic vesicles in many different cell types, such as breast cancer cells [26], GC cells [27], and prostate cancer cells [28]. However, studies exploring autophagy in clinical tumor samples are scarcely reported.

In the present study, we used TEM to determine the difference in autophagy levels between GC and adjacent NNM tissues and have, for the first time, shown that GC tissues exhibit higher autophagic activity. Indeed, our results indicate that autophagy may be an important marker for distinguishing GC from adjacent NNM tissue. Nevertheless, we have only analyzed 8 samples with the TEM assay in this study, which is a low number of samples to have statistically significant results. In future studies, we will try to collect more fresh samples for the TEM assay to determine the difference in autophagy levels between GC and adjacent NNM tissues.

Beclin-1 is an important initiator of autophagy [7]. The association between autophagy and Beclin-1 has been well established [23], and it has been used in many studies as a marker to monitor autophagy [8, 9]. Furthermore, it has been demonstrated that acetylation of Beclin-1 can lead to inhibition of autophagic responses [23]. Indeed, knockdown of Beclin-1 in GC cells results in inhibition of autophagy, which in turn promotes EMT and metastasis [29]. Immunohistochemistry is another indispensable tool for the evaluation of autophagy in situ [30]. For example, expression of Beclin-1 and its role autophagy in a variety of tumors has been explored with no definite conclusions [11, 15]. Compared with corresponding normal tissues, Beclin-1 expression was reported to be low in hepatocellular carcinoma [15] and colorectal cancer [16]. Contrary to these findings, in intrahepatic cholangiocarcinoma [14], ovarian epithelial carcinoma [10], and nasopharyngeal carcinoma [13], Beclin-1 expression was higher compared to corresponding normal tissue. Contradictory findings have been reported in the literature regarding Beclin-1 expression in GC [31]. In a study by Zhou et al. [11], Beclin-1 expression was reportedly lower in tumor tissues compared to corresponding normal tissue, while Yu et al. [12] reported opposing findings. In the present study, we have demonstrated that Beclin-1 expression was significantly higher in GC tissues indicating that GC tissues exhibit more autophagic activity. In this study, we have examined autophagy using TEM, and by examining the expression of Beclin-1 using immunohistochemistry. Nevertheless, other techniques and molecular markers [9] could be utilized to monitor autophagy in future studies and further in vitro, and animal models are clearly needed.

In the present study, we have also examined the prognostic role of Beclin-1 in GC. Previous studies have reported the prognostic role of Beclin-1 in other human tumors, but the exact role of Beclin-1 still remains to be defined. For example, high Beclin-1 expression was considered to be a marker of poor prognosis in nasopharyngeal carcinoma [13] and ovarian epithelial carcinoma [10]. However, other studies have reported that high Beclin-1 expression was related to good prognosis in patients with tumors such as intrahepatic cholangiocarcinoma [14], colorectal cancer [16], and hepatocellular carcinoma [15]. Zhou et al. (2012) [11] and Yu et al. (2013) [12] reported that low expression of Beclin-1 predicted adverse OS and RFS of GC patients, indicating that Beclin-1 was a marker of better prognosis in GC. Contrary to this, Masuda et al. (2016) [32] reported that autophagy markers, including Beclin-1, were associated with poor survival of patients with GC. In this study, high expression of Beclin-1 was significantly correlated with shorter OS and RFS, and Beclin-1 was found to be a marker of poor prognosis in GC.

SIRT1 contributes to cancer cell death by inhibiting tumor growth; however, it also supports cancer cell growth and survival by upregulating oncogenic signaling pathways [17]. SIRT1 expression in GC varies according to tumor type, the tumor microenvironment, and cellular stress, and the role of SIRT1 in GC progression is still not fully understood. In some studies, SIRT1 was found to be highly expressed in GC tissues compared to adjacent NNM and was reported as a poor prognostic indicator in GC [18, 19]. Contrary to this, Yang et al. (2013) [20] found that SIRT1 was downregulated in GC tissues, leading to the inhibition of GC, by inducing G1 phase arrest through the NF-kB/cyclin D1 pathway. In a study by Cha et al. (2009) [18], SIRT1 was highly expressed in GC tissues, but was associated with a better prognosis. In our study, SIRT1 was highly expressed in GC tissues, compared to adjacent NNM, which is in accordance with findings of other published studies. It was also a poor prognostic factor significantly associated with shorter OS and RFS.

It has been reported that SIRT1 increases cell invasion, anoikis resistance, and EMT of GC cells [21]. Resveratrol, an agonist of SIRT1, was found to inhibit GC cell growth and induce G1 phase arrest and senescence in a SIRT1-dependent manner [22]. Collectively, these studies indicated that SIRT1 is involved in both suppression and promotion of GC cell growth.

Many studies have shown that SIRT1 has a close relationship with autophagy in several physiological and pathological processes [23, 33, 34]. The role of SIRT1 in tumors is significantly heterogeneous. In prostate cancer, SIRT1-mediated autophagy plays a role in modulating angiogenesis and cellular senescence [35], while in human breast adenocarcinoma MCF7 cells, it promoted autophagosome maturation and tumor growth [23]. It has also been reported that SIRT1 could promote autophagosome maturation by deacetylating Beclin-1 [23]. In our previously published study, we showed that SIRT1 regulates autophagy through the SIRT1-FoxO1-Rab7 axis, deacetylation of ATGs 5, 7, and 8 and other mediators, such as H4K16ac, FoxO3, E2F1, and p73 [17]. Recently, Ramalinga et al. (2015) [35] demonstrated that miR-212 inhibits autophagy by inhibiting SIRT1 expression in prostate cancer cells, while transfection of cells with SIRT1 induced autophagy. In addition, SIRT1 could reduce polyglutamine cellular toxicity by inducing autophagy in SH-SY5Y cells [36]. So far, there have been no studies examining the relationship between the SIRT1 and autophagy in GC. In this study, we have examined the expression of SIRT1 and autophagy marker Beclin-1 in GC tissues, and our results showed that SIRT1 expression was significantly positively correlated with Beclin-1 expression. In addition, patients with Beclin-1/SIRT1 high/high expression had the shortest OS and RFS. These results point to an association between SIRT1 and autophagy and we confirmed that SIRT1 may promote the development of GC by regulating autophagy.

In conclusion, this study is the first to demonstrate that GC tissues exhibit higher levels of autophagic activity compared to adjacent NNM tissues and that Beclin-1 and SIRT1 expression can be used as novel prognostic indicators in GC patients. In addition, SIRT1 expression was positively correlated with Beclin-1 expression, which is an important autophagy marker. Collectively, these results suggest a close link between SIRT1 and autophagy which may be involved in the progression of GC and may be used as a new therapeutic target for the treatment of GC. Nevertheless, further in vitro and in vivo studies are needed to establish the exact role and molecular mechanism of SIRT1-autophagy axis in the progression of GC.

## Supplementary Material

Table S1: The clinicopathological data of the 8 patients selected for TEM assay was collected in 2013. Patient age ranged from 27 to 74 years (median, 51.7 ± 15.0 years), 5 were male and 3 were female, and the other clinicopathological data (height, weight, BMI, tumor size, tumor location, pathological type, lauren classification, histologic grade, lymph node metastasis, tumor invasion, TNM stage, vascular cancer embolus) was show in Table S1.
Table S2: The Pearson's χ^2^ test was used to examine the expression of Beclin-1 and SIRT1 expression between NNM and cancer tissues. High expression of Beclin-1 was detected in 24 out of 96 adjacent NNM tissues, and 57/96 cancer tissues; high SIRT1 expression was detected in 19.8% adjacent NNM tissues and 55.2% cancer tissues. Both the expression of Beclin-1 and SIRT1 between NNM and cancer tissues was found to be statistically significant.

## Figures and Tables

**Table 1:** Association of Beclin-1, SIRT1, and their combined expression status with clinicopathological characteristics in GC.

Characteristics	*N*	Beclin-1 expression		SIRT1 expression		Beclin-1/SIRT1 expression				
		High (%)	*p*	High (%)	*p*	Low/low (%)	Low/high (%)	High/low (%)	High/high (%)	*p*
Gender										
Female	21	13 (61.9)	0.789	11 (52.4)	0.768	8 (38.1)	0 (0.0)	2 (9.5)	11 (52.4)	0.341
Male	75	44 (58.7)		42 (56.0)		22 (29.3)	9 (12.0)	11 (14.7)	33 (44.0)	
Age (y)										
<60	45	24 (53.3)	0.258	20 (44.4)	0.046^*∗*^	19 (42.2)	2 (4.4)	6 (13.3)	18 (40.0)	0.110
≥60	51	33 (64.7)		33 (64.7)		11 (21.6)	7 (13.7)	7 (13.7)	26 (51.0)	
BMI										
<24	75	43 (57.3)	0.441	39 (52.0)	0.232	25 (33.3)	7 (9.3)	11 (14.7)	32 (42.7)	0.680
≥24	21	14 (66.7)		14 (66.7)		5 (23.8)	2 (9.5)	2 (9.5)	12 (57.1)	
Tumor size (cm)										
≤5	66	32 (48.5)	0.001^*∗*^	31 (47.0)	0.016^*∗*^	25 (37.9)	9 (13.6)	10 (15.2)	22 (33.3)	0.002^*∗*^
>5	30	25 (83.3)		22 (73.3)		5 (16.7)	0 (0.0)	3 (10.0)	22 (73.3)	
Tumor location										
Up	36	23 (63.9)	0.248	17 (47.2)	0.232	12 (33.3)	1 (2.8)	7 (19.4)	16 (44.4)	0.222
Middle	15	6 (40.0)		7 (46.7)		6 (40.0)	3 (20.0)	2 (13.3)	4 (26.7)	
Lower	45	28 (62.2)		29 (64.4)		12 (26.7)	5 (11.1)	4 (8.9)	24 (53.3)	
Lauren classification										
Intestinal	60	32 (53.3)	0.120	31 (51.7)	0.368	22 (36.7)	6 (10.0)	7 (11.7)	25 (41.7)	0.441
Diffuse	36	25 (69.4)		22 (61.1)		8 (22.2)	3 (8.3)	6 (16.7)	19 (52.8)	
Histologic grade										
WD and MD	27	8 (39.6)	<0.001^*∗∗*^	9 (33.3)	0.007^*∗*^	16 (59.3)	3 (11.1)	2 (7.4)	6 (22.2)	0.001^*∗*^
PD	69	49 (71.0)		44 (63.8)		14 (20.3)	6 (8.7)	11 (15.9)	38 (55.1)	
LN metastasis										
Absence	24	3 (12.5)	<0.001^*∗∗*^	3 (12.5)	<0.001^*∗∗*^	19 (79.2)	2 (8.3)	2 (8.3)	1 (4.2)	<0.001^*∗∗*^
Presence	72	54 (75.0)		50 (69.4)		11 (15.3)	7 (9.7)	11 (15.3)	43 (59.7)	
Tumor invasion										
EGC	9	4 (44.4)	0.547	1 (11.1)	0.015^*∗*^	5 (55.6)	0 (0.0)	3 (33.3)	1 (11.1)	0.026^*∗*^
AGC	87	53 (60.9)		52 (59.8)		25 (28.7)	9 (10.3)	10 (11.5)	43 (49.4)	
TNM stage										
I and II	23	3 (13.0)	<0.001^*∗∗*^	4 (17.4)	<0.001^*∗∗*^	17 (73.9)	3 (13.0)	2 (8.7)	1 (4.3)	<0.001^*∗∗*^
III and IV	73	54 (74.0)		49 (67.1)		13 (17.8)	6 (8.2)	11 (15.1)	43 (58.9)	
VCE										
Absence	57	28 (49.1)	0.013^*∗*^	28 (49.1)	0.147	24 (42.1)	5 (8.8)	5 (8.8)	23 (40.4)	0.030^*∗*^
Presence	39	29 (74.4)		25 (64.1)		6 (15.4)	4 (10.3)	8 (20.5)	21 (53.8)	
SIRT1 expression										
Low	43	13 (30.2)	<0.001^*∗∗*^							
High	53	44 (83.0)								

BMI, body mass index; LN metastasis, lymph node metastasis; TNM stage, tumor node metastasis stage; VCE, vascular cancer embolus; WD, well differentiation; MD, middle differentiation; PD, poor differentiation; EGC, early gastric cancer; AGC, advantage gastric cancer; *N*, number of patients; ^*∗*^ *p* < 0.05; ^*∗∗*^ *p* < 0.001.

**Table 2:** Multivariate analysis of the related factors of the Beclin-1 and SIRT1 high expression in GC.

Related factors	OR	95% CI	*p*
*High Beclin-1 expression*			
Tumor size (>5 versus ≤5 cm)	7.211	(1.382, 37.632)	0.019^*∗*^
Histologic grade (PD versus WD and MD)	2.020	(0.495, 8.249)	0.327
LN metastasis (Positive versus Negative)	5.567	(0.548, 56.539)	0.147
TNM stage (III and IV versus I and II)	3.156	(0.416, 23.917)	0.266
VCE (Positive versus Negative)	1.682	(0.502, 5.635)	0.399
SIRT1 expression (High versus Low)	4.617	(1.416, 15.053)	0.011^*∗*^
*High SIRT1 expression*			
Age (≥60 versus <60 y)	3.016	(0.969, 9.387)	0.057
Tumor size (>5 versus ≤5 cm)	1.564	(0.430, 5.691)	0.498
Histologic grade (PD versus WD and MD)	0.978	(0.274, 3.492)	0.973
LN metastasis (Positive versus Negative)	7.261	(0.657, 80.181)	0.106
Tumor invasion (AGC versus EGC)	9.835	(0.607, 159.346)	0.108
TNM stage (III and IV versus I and II)	0.469	(0.055, 3.988)	0.488
Beclin1 expression (High versus Low)	7.818	(1.987, 30.756)	0.003^*∗*^

LN metastasis, lymph node metastasis; TNM stage, tumor node metastasis stage; VCE, vascular cancer embolus; WD, well differentiation; MD, middle differentiation; PD, poor differentiation; EGC, early gastric cancer; AGC, advantage gastric cancer; OR, odd ratio; CI, confidence interval; ^*∗*^ *p* < 0.05.

**Table 3:** Univariate Cox proportional hazards regression analysis of GC patients' clinicopathological characteristics and OS and RFS.

Characteristics	*N*	OS		RFS	
		HR (95% CI)	*p*	HR (95% CI)	*p*
BMI (≥24 versus <24)	21 versus 75	0.543 (0.274–1.072)	0.079	0.553 (0.280–1.094)	0.089
Tumor size (>5 versus ≤5cm)	30 versus 66	1.745 (1.025–2.969)	0.040^*∗*^	1.710 (1.005–2.911)	0.048^*∗*^
Lauren classification (Diffuse versus Intestinal)	36 versus 60	1.373 (0.817–2.307)	0.231	1.360 (0.809–2.286)	0.245
Histologic grade (PD versus WD and MD)	69 versus 27	2.673 (1.383–5.167)	0.003^*∗*^	2.663 (1.377–5.150)	0.004^*∗*^
LN metastasis (Positive versus Negative)	72 versus 24	49.924 (6.792–366.946)	<0.001^*∗∗*^	46.792 (6.380–343.202)	<0.001^*∗∗*^
Tumor invasion (AGC versus EGC)	87 versus 9	27.242 (1.198–619.630)	0.038^*∗*^	26.825 (1.152–624.503)	0.041^*∗*^
TNM stage (III and IV versus I and II)	72 versus 24	37.857 (5.199–275.640)	<0.001^*∗∗*^	36.784 (5.056–267.642)	<0.001^*∗∗*^
VCE (Positive versus Negative)	39 versus 57	1.551 (0.927–2.596)	0.095	1.540 (0.920–2.577)	0.100
SIRT1 expression (High versus Low)	53 versus 43	5.163 (2.820–9.452)	<0.001^*∗∗*^	4.949 (2.706–9.052)	<0.001^*∗∗*^
Beclin1 expression (High versus Low)	57 versus 39	5.994 (3.055–11.763)	<0.001^*∗∗*^	6.019 (3.069–11.804)	<0.001^*∗∗*^
Beclin-1/SIRT1 expression (Low/High versus Low/Low)	9 versus 30	2.926 (0.846–10.117)	0.090	2.911 (0.841–10.081)	0.092
Beclin-1/SIRT1 expression (High/Low versus Low/Low)	13 versus 30	1.980 (1.183–3.314)	0.009^*∗*^	2.006 (1.198–3.358)	0.008^*∗*^
Beclin-1/SIRT1 expression (High/High versus Low/Low)	44 versus 30	2.297 (1.718–3.071)	<0.001^*∗∗*^	2.266 (1.697–3.025)	<0.001^*∗∗*^
Beclin-1/SIRT1 expression (High/Low versus Low/High)	13 versus 9	1.280 (0.383–4.282)	0.688	1.416 (0.424–4.723)	0.572
Beclin-1/SIRT1 expression (High/High versus Low/High)	44 versus 9	1.870 (1.110–3.149)	0.019^*∗*^	1.889 (1.123–3.178)	0.017^*∗*^
Beclin-1/SIRT1 expression (High/High versus High/Low)	44 versus 13	3.051 (1.409–6.604)	0.005^*∗*^	2.853 (1.322–6.154)	0.008^*∗*^

BMI, body mass index; LN metastasis, lymph node metastasis; TNM stage, tumor node metastasis stage; VCE, vascular cancer embolus; WD, well differentiation; MD, middle differentiation; PD, poor differentiation; EGC, early gastric cancer; AGC, advantage gastric cancer; *N*, number of patients; OS, overall survival; RFS, relapse-free survival; HR, hazard ratio; CI, confidence interval; ^*∗*^ *p* < 0.05; ^*∗∗*^ *p* < 0.001.

**Table 4:** Multivariate Cox regression analysis for OS and RFS.

Characteristics	*N*	OS		RFS	
		HR (95% CI)	*p*	HR (95% CI)	*p*
LN metastasis (positive versus negative)	72 versus 24	27.346 (3.572–209.337)	0.001^*∗*^	25.195 (3.293–192.743)	0.002^*∗*^
SIRT1 expression (high versus low)	53 versus 43	2.393 (1.273–4.499)	0.007^*∗*^	2.230 (1.187–4.191)	0.013^*∗*^
Beclin1 expression (high versus low)	57 versus 39	2.131 (1.057–4.297)	0.034^*∗*^	2.237 (1.108–4.518)	0.025^*∗*^

LN metastasis, lymph node metastasis; *N*, number of patients; OS, overall survival; RFS, relapse-free survival; HR, hazard ratio; CI, confidence interval; ^*∗*^ *p* < 0.05.
