# Supplementary material for: The Prognostic Role of SIRT1-Autophagy Axis in Gastric Cancer
Source: Dis Markers. 2016 Dec 14;2016:6869415. doi: 10.1155/2016/6869415 (PMC5192295; doi:10.1155/2016/6869415)
Supplement: Supplementary file 1 — Table S1: The clinicopathological data of the 8 patients selected for TEM assay was collected in 2013. Patient age ranged from 27 to 74 years (median, 51.7 ± 15.0 years), 5 were male and 3 were female, and the other clinicopathological data (height, weight, BMI, tumor size, tumor location, pathological type, lauren classification, histologic grade, lymph node metastasis, tumor invasion, TNM stage, vascular cancer embolus) was show in Table S1. Table S2: The Pearson's χ2 test was used to examine the expression of Beclin-1 and SIRT1 expression between NNM and cancer tissues. High expression of Beclin-1 was detected in 24 out of 96 adjacent NNM tissues, and 57/96 cancer tissues; high SIRT1 expression was detected in 19.8% adjacent NNM tissues and 55.2% cancer tissues. Both the expression of Beclin-1 and SIRT1 between NNM and cancer tissues was found to be statistically significant. [file 6869415.f1.pdf]

Table S1. The clinicopathological data of the 8 patients selected for TEM assay

Characteristics	Patient number							
	**1**	**2**	**3**	**4**	**5**	**6**	**7**	**8**
**Gender**	M	M	M	M	F	F	F	M
**Age(y)**	46	42	67	74	27	50	47	61
**Height (cm)**	168	163	165	160	153	163	168	160
**Weight (kg)**	65	54	69	60	53	60	56	55
**BMI**	23.03	20.32	25.34	23.44	22.64	22.58	19.84	21.48
**Tumor size(cm)**	4.8	5.4	8.3	4.5	3.1	5.2	4.6	7.2
**Tumor location**	L	L	UM	L	L	L	L	L
**Pathological type**	AC	AC	AC	AC	AC	AC	AC	AC
**Lauren classification**	Int	Int	Int	Int	Dif	Int	Dif	Dif
**Histologic grade**	PD	PD	MD	PD	PD	PD	MD	PD
**LN metastasis (NMLN / NDLN)**	Pre (1/23)	Pre (11/26)	Ab (0/20)	Pre (10/24)	Ab (0/32)	Pre (15/46)	Pre (12/45)	Pre (17/40)
**Tumor invasion**	AGC	AGC	AGC	AGC	AGC	AGC	AGC	AGC
**TNM stage**	IIIA	IIIC	IIB	IIIC	IIB	IIIC	IIIC	IIIC
**VCE**	Ab	Pre	Pre	Ab	Ab	Ab	Pre	Pre

Abbreviations: M, Male; F, Female; BMI, body mass index; L, Lower; UM, Up and Middle; AC, Adenocarcinoma; Int, Intestinal; Dif, Diffuse; MD, middle differentiation; PD, poor differentiation; LN metastasis, lymph node metastasis; NMLN, Number of metastatic lymph nodes; NDLN, Number of dissected lymph nodes; Pre, Presence; Ab, Absence; AGC, advantage gastric cancer; TNM stage, tumor node metastasis stage; VCE, vascular cancer embolus.
