# Supplementary material for: The Prognostic Role of SIRT1-Autophagy Axis in Gastric Cancer
Source: Dis Markers. 2016 Dec 14;2016:6869415. doi: 10.1155/2016/6869415 (PMC5192295; doi:10.1155/2016/6869415)
Supplement: Supplementary file 2 [file 6869415.f2.pdf]

Table S2. Beclin-1 and SIRT1 expression in 96 patients with GC and adjacent NNM

Histological types	N	Beclin-1 expression		SIRT1 expression	
		High (%)	*p*	High (%)	*p*
**Adjacent NNM**	96	24(25.0)	< 0.001**	19(19.8)	< 0.001**
**Cancer**	96	57(59.4)		53(55.2)	

Abbreviations: Adjacent NNM, adjacent non-neoplåastic mucosa; N, number of patients; ***p* < 0.001.
